# Unraveling the Gas-Sensing Mechanisms of Lead-Free Perovskites Supported on Graphene

**DOI:** 10.1021/acssensors.2c01581

**Published:** 2022-11-21

**Authors:** Juan Casanova-Chafer, Rocio Garcia-Aboal, Pedro Atienzar, Eduard Llobet

**Affiliations:** † MINOS Research Group, Department of Electronics Engineering, Universitat Rovira i Virgili, 43007Tarragona, Spain; ‡ Instituto de Tecnología Química (Universitat Politècnica de València − Consejo Superior de Investigaciones Científicas), 46022Valencia, Spain

**Keywords:** lead-free perovskite, nanocrystal, graphene, gas sensor, sensing mechanism

## Abstract

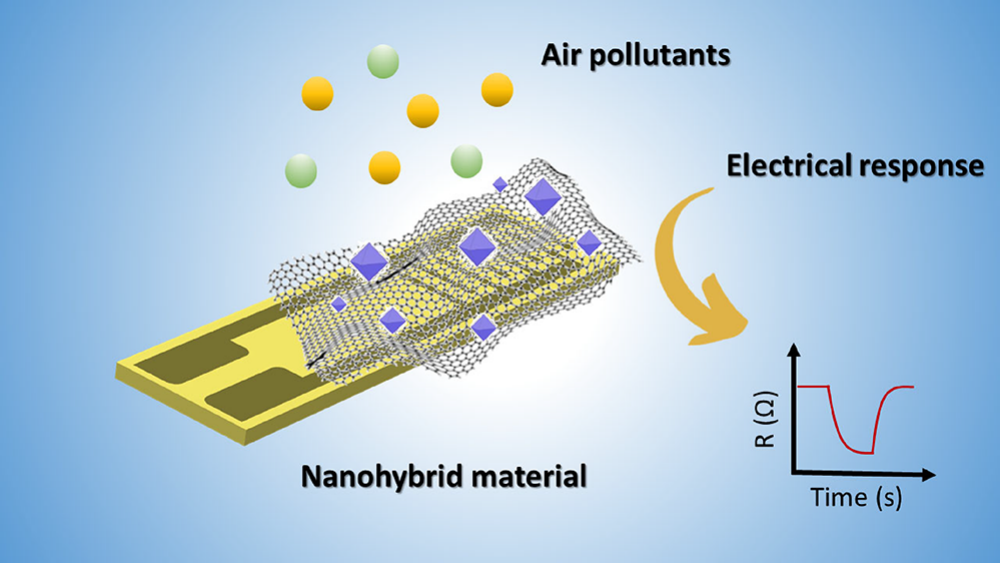

Lead halide perovskites have been attracting great attention due to their outstanding properties and have been utilized for a wide variety of applications. However, the high toxicity of lead promotes an urgent and necessary search for alternative nanomaterials. In this perspective, the emerging lead-free perovskites are an environmentally friendly and harmless option. The present work reports for the first time gas sensors based on lead-free perovskite nanocrystals supported on graphene, which acts as a transducing element owing to its high and efficient carrier transport properties. The use of nanocrystals enables achieving excellent sensitivity toward gas compounds and presents better properties than those of bulky perovskite thin films, owing to their quantum confinement effect and exciton binding energy. Specifically, an industrially scalable, facile, and inexpensive synthesis is proposed to support two different perovskites (Cs_3_CuBr_5_ and Cs_2_AgBiBr_6_) on graphene for effectively detecting a variety of harmful pollutants below the threshold limit values. H_2_ and H_2_S gases were detected for the first time by utilizing lead-free perovskites, and ultrasensitive detection of NO_2_ was also achieved at room temperature. In addition, the band-gap type, defect tolerance, and electronic surface traps at the nanocrystals were studied in detail for understanding the differences in the sensing performance observed. Finally, a comprehensive sensing mechanism is proposed.

Air pollution, defined as the release of harmful gases into the atmosphere, is becoming a major societal concern due to its effects on human health and the environment. In addition to the acute exposure to gases in leakage events, long-term exposure to low concentrations of pollutants can induce significant respiratory and cardiovascular health issues, resulting in millions of premature deaths yearly.^1^ This exposure to trace levels of gases is significantly dangerous considering that many are colorless and odorless. Therefore, there is a growing demand for novel miniaturized affordable and reliable devices that are able to detect pollutants at trace levels. Devices that are able to implement real-time monitoring of the air quality for raising an alarm when pollutants exceed the threshold limit values (TLVs) are much sought. In this sense, the environmental monitoring market is estimated to grow at a compound annual growth rate (CAGR) of 4.5% to reach USD 17.98 billion by 2026.^2^ Within this market, the air quality monitoring market size was valued at over 3.5B in 2018 and is expected to grow at a 7% CAGR. In consequence, to achieve large gas-sensor networks, there is a growing demand for developing cost-effective devices. Despite some instrumental techniques as gas chromatography or surface-enhanced Raman spectroscopy (SERS) being extremely sensitive, selective, and accurate, significant drawbacks are preventing their widespread distribution over large areas. This is because these techniques are bulky and costly and require trained personnel, and real-time monitoring is still a challenge.^3,4^

Within the different sensing devices, chemical resistive sensors (chemoresistors) have emerged as an interesting technology due to their ease of use, inexpensiveness, stability, and suitability for miniaturization and implementing real-time monitoring.^5^ With all of that, chemoresistors are interesting candidates for developing the new generation of unattended and ubiquitous gas-sensing networks. However, for a feasible spatial distribution of battery-powered monitoring nodes, low-power consumption devices, which can be achieved by developing room-temperature sensors, are needed. Chemoresistors based on metal oxides (MOXs) have received large research efforts during the last decades owing to their high sensitivity and fast adsorption/desorption of gas molecules dynamics.^6^ However, MOX gas sensors usually require high operating temperatures to activate their sensing properties and show limited selectivity,^7^ which can prevent their effective implementation in commercial devices. The reason is that the high temperatures needed significantly increase the energy consumption and increase the fabrication costs since heating elements and temperature controllers are required. Additionally, high operating temperatures can compromise the long-term stability and repeatability, owing to phase, morphological, or even compositional changes in the nanostructures. For instance, coalescence effects on MOXs can modify the structures, resulting in their agglomeration in larger entities and therefore altering their sensing performance.

Thereby, recent research efforts have been focused on the development of sensitive layers that are able to be operated on under room-temperature conditions. Within the different available nanomaterials, graphene has emerged as a promising candidate owing to its outstanding properties. For instance, graphene virtually shows the highest surface area-to-volume ratio, resulting in a larger area exposed to the environment for interacting with gas compounds. In addition, graphene shows low noise levels owing to the high carrier density and mobility, while its synthesis at an industrial scale has an affordable production cost.^8^ Conversely, pristine graphene tends to show significant drawbacks that are preventing its effective implementation in commercial devices. Its rather inert surface and poor specificity result in low sensitivity and selectivity, respectively. Within this perspective, graphene surface modification is a feasible strategy for improving sensing performance. Different approaches as graphene decoration with metal or metal oxide nanoparticles have been shown to be good options for enhancing sensitivity and, to some extent, selectivity.^5^ However, the resulting hybrid nanomaterials usually require applying relatively high operating temperatures, challenging the capability of graphene to work under room conditions.

Alternatively, nanomaterials such as lead halide perovskite nanocrystals have been used for decorating graphene and consequently improving sensing performance.^9^ Like graphene, these nanocrystals can be used for sensing purposes at room temperature, revealing a suitable alignment between the requirements of both nanomaterials. In this sense, the creation of hybrids combining graphene and perovskite nanocrystals led to low-power and inexpensive gas sensors owing to their room-temperature operation. Furthermore, their combination presents an interesting synergistic effect.^10^ On the one hand, the high chemical reactivity of halide perovskite improves the low sensitivities obtained using pristine graphene, and on the other hand, the instability of halide perovskites in the presence of ambient moisture is well known,^11^ which results in a fast degradation. However, the high hydrophobic character of graphene can protect the perovskite nanocrystals against ambient moisture, increasing their stability and enabling their use in ambient monitoring applications. In addition, the use of perovskite nanocrystals presents better optical and electrical properties compared to the bulk phase. This is due to their tunable size and high quantum confinement.^12^ Nevertheless, despite the outstanding sensing performance in using halide perovskites, most of them use lead (Pb) as a divalent cation. Their manipulation and use in synthesis protocols involve severe risks to human health and detrimental environmental effects. Lead exposure, even to low concentrations, can significantly affect the cardiovascular and neurological systems.^13^ Therefore, the use of lead is progressively restricted by the authorities, even in electrical and electronic systems. In consequence, despite the noteworthy properties of lead halide perovskites, their effective implementation in commercial devices is limited.

For that reason, very recently, some research efforts have redirected toward the use of lead-free halide perovskites for reducing the potential dangerousness and retaining their excellent properties. This type of perovskites has been reported for a wide variety of applications such as photodetectors, solar cells, and LEDs.^14^ Nevertheless, this paper reports for the first time a comprehensive gas-sensing study of nanohybrids based on different lead-free perovskites supported on graphene. A green-chemistry and environmentally friendly approach is proposed for developing durable, reproducible sensitive gas sensors using novel perovskites. Two perovskite NCs have been studied: (a) Cs_3_Cu_2_Br_5_ characterized by its direct band gap, outstanding stability, and nontoxicity of Cu(I) and (b) Cs_2_AgBiBr_6_ with its indirect band gap, nontoxic character, and remarkable thermal and environmental stability.^15,16^ These perovskites were supported on graphene for detecting a wide variety of gas compounds such as NO_2_, NH_3_, H_2_S, and H_2_. As a result, the highest sensing performance of lead-free perovskites (i.e., as thin films or nanocomposites) was obtained to date. In addition, the graphene-supported lead-free perovskites show even higher gas-sensing performance than that of graphene-supported lead halide perovskites. Therefore, the method proposed leverages the high versatility of perovskites and their straightforward synthesis and integration into transducing substrates, which can be easily exported to a large-production scale.

## Experimental Section

### Synthesis of Lead-Free Nanocrystals and Suspension on Graphene

Two lead-free perovskite nanocrystals (Cs_3_CuBr_5_ and Cs_2_AgBiBr_6_) were synthesized through hot injection methods.^17,18^ Both synthesis processes required a first step for obtaining the cesium oleate, which was prepared by adding 0.814 g of Cs_2_CO_3_, 2.5 mL of oleic acid (OA), and 40 mL of octadecene (ODE) in a 100 mL three-neck flask. The mixture was kept at 120 °C under vacuum for 1 h, and afterward, the temperature was increased to 150 °C under a nitrogen atmosphere for achieving the complete solubilization of Cs_2_CO_3_. Then, the Cs–oleate solution was cooled down until room temperature by switching off the hot plate, resulting in the precipitation of the Cs–oleate and the ODE as a supernatant. In the next synthesis steps, the Cs–oleate will be preheated to 100 °C before injection.

Cs_3_Cu_2_Br_5_ NCs were synthesized by adding 71 mg of CuBr_2_ to 6 mL of ODE. The solution was heated at 120 °C for 1 h under vacuum, and subsequently, 1 mL of OA and 1 mL of dried oleylamine (OLA) were added under a nitrogen atmosphere. When the complete solubilization of CuBr_2_ was achieved, the temperature was increased to 160 °C, and 6 mL of the Cs–oleate previously obtained was quickly injected. After 5 s, the reaction mixture was cooled down using an ice-water bath, inducing the nanocrystals’ precipitation. Finally, a centrifugation process (6000 rpm for 5 min) was performed for extracting the nanocrystals, which were redispersed in isopropanol, resulting in a stable perovskite solution.

Cs_2_AgBiBr_6_ NCs were obtained by adding BiBr_3_ (45 mg), AgNO_3_ (17 mg), and HBr (100 μL) to 4 mL of ODE, and the solution was heated under vacuum for 1 h at 120 °C. Once the complete solubilization was achieved, the solution was further heated up to 200 °C under a nitrogen flow and 0.8 mL of the Cs–oleate was injected. Afterward, the mixture was cooled by using an ice-water bath after 5 s. The NCs were obtained after a centrifugation process (7000 rpm for 10 min) and were finally redispersed in isopropanol and washed several times.

After the lead-free perovskite synthesis, commercially available graphene nanoplatelets (Strem Chemicals Inc., US) were dispersed in isopropanol (0.3 mg/mL). Then, graphene was exfoliated by applying a pulsed sonication (1 s on/2 s off) at 280 W for 90 min on an ultrasonic tip. Afterward, Cs_3_Cu_2_Br_5_ and Cs_2_AgBiBr_6_ NCs were added (5% wt.) to graphene solutions. For achieving a suitable suspension of perovskite NCs on graphene, the mixtures were placed in an ultrasonic bath for 1 h. The presence of defects and surface oxygenated functional groups in the graphene probably promotes noncovalent interactions (i.e., Van der Waals forces and hydrogen bonds) with the perovskite NCs.

### Sensor Fabrication and Gas-Sensing Setup

The resulting graphene was loaded with the different lead-free perovskite NCs deposited by a spray coating technique and employing pure nitrogen as a carrier gas. Specifically, the hybrid nanomaterials were deposited onto interdigitated electrodes (IDE) already screen-printed in alumina substrates. The sensing devices were placed in an airtight Teflon chamber (35 cm^3^ of volume), which is connected to a gas mixing and delivery system. A pure dry air atmosphere (Air Premier purity: 99.999%) and calibrated gas cylinders containing the target gas diluted in dry air were utilized in the sensing measurements. Additionally, the effect of the ambient moisture was also studied, and for this reason, a controller evaporator mixer (Bronkhorst High-Tech B.V., Netherlands) was used to humidify the atmosphere.

It is worth noting that, during all the measurements, a low flow rate was applied (100 mL/min) using a set of mass-flow controllers (Bronkhorst High-Tech B.V., Netherlands) and electronic valves. The gas sensors were stabilized for 15 min under dry air before each exposition to a specific concentration of the target gas for 5 min. Indeed, successive and increasing concentrations were applied by performing their dilution with dry air. Then, sensor responses were defined as Δ*R*/*R*_0_ expressed in percentages, where Δ*R* is the resistance change recorded over gas exposures, while *R*_0_ is given by the baseline resistance of the sensor in air.

## Results and Discussion

### Material Characterization

**Figure 1:**
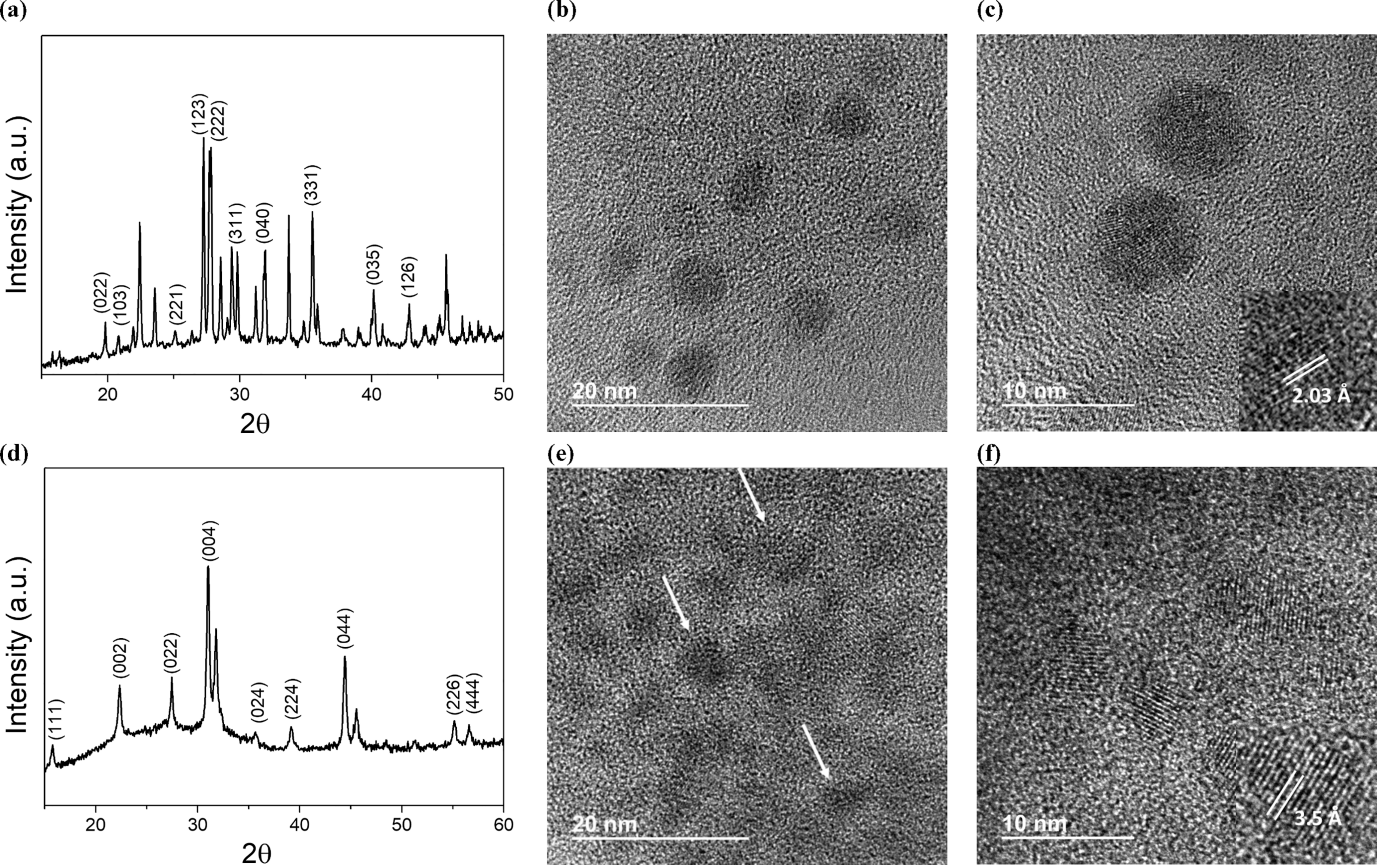
XRD patterns of Cs_3_Cu_2_Br_5_ (a) and Cs_2_AgBiBr_6_ (d) perovskite nanocrystals. HR-TEM images at different magnification values for Cs_3_Cu_2_Br_5_ (b, c) and Cs_2_AgBiBr_6_ (e, f) nanocrystals.

The X-ray diffraction (XRD) patterns of the perovskite NCs were recorded on a Philips X’PERT diffractometer that was equipped with a proportional detector and a secondary graphite monochromator. The data were collected stepwise over the range 2θ = 2–20° at steps of 0.02° at an accumulation time of 20 s/step and using the Cu Kα radiation (λ = 1.54178 Å). Figure 1a,d shows the diffractograms obtained for Cs_3_Cu_2_Br_5_ and Cs_2_AgBiBr_6_ NCs, respectively. The identified XRD patterns were compared with those previously reported,^17−19^ revealing a good match and confirming the orthorhombic and the cubic phase for the Cs_3_Cu_2_Br_5_ and Cs_2_AgBiBr_6_, respectively.

High-resolution transmission electron microscope (HRTEM) images were recorded using a JEOL JEM 2100F, and the average nanocrystal size and interplanar distances were obtained through ImageJ and Gatan software. Figure 1b–f shows some examples of both perovskites at different magnifications. Interestingly, both nanocrystals present similar diameters, that is, 6.7 ± 1.6 and 4.8 ± 1.4 nm for Cs_3_Cu_2_Br_5_ and Cs_2_AgBiBr_6_, respectively. Likewise, interplanar distances of 2.03 and 3.5 Å were registered for Cs_3_Cu_2_Br_5_ and Cs_2_AgBiBr_6_, respectively. These similar parameters and high crystallinity observed for both types of nanocrystals would enable a reliable comparison of the sensing performance. The nanocrystal size distribution is illustrated in Figure S1, and an image of the bare graphene nanoplatelets is shown in Figure S2. Finally, the resulting nanomaterial, which consists of lead-free perovskites supported on graphene, was analyzed through a field-effect scanning electron microscope (FESEM). Figure S3a shows a homogeneous layer of the nanohybrid developed. It is worth noting that the porous surface is of paramount importance for the gas sensing performance.^20^ The bright spots in Figure S3b reveal the random nanocrystal distribution on graphene.

**Figure 2:**
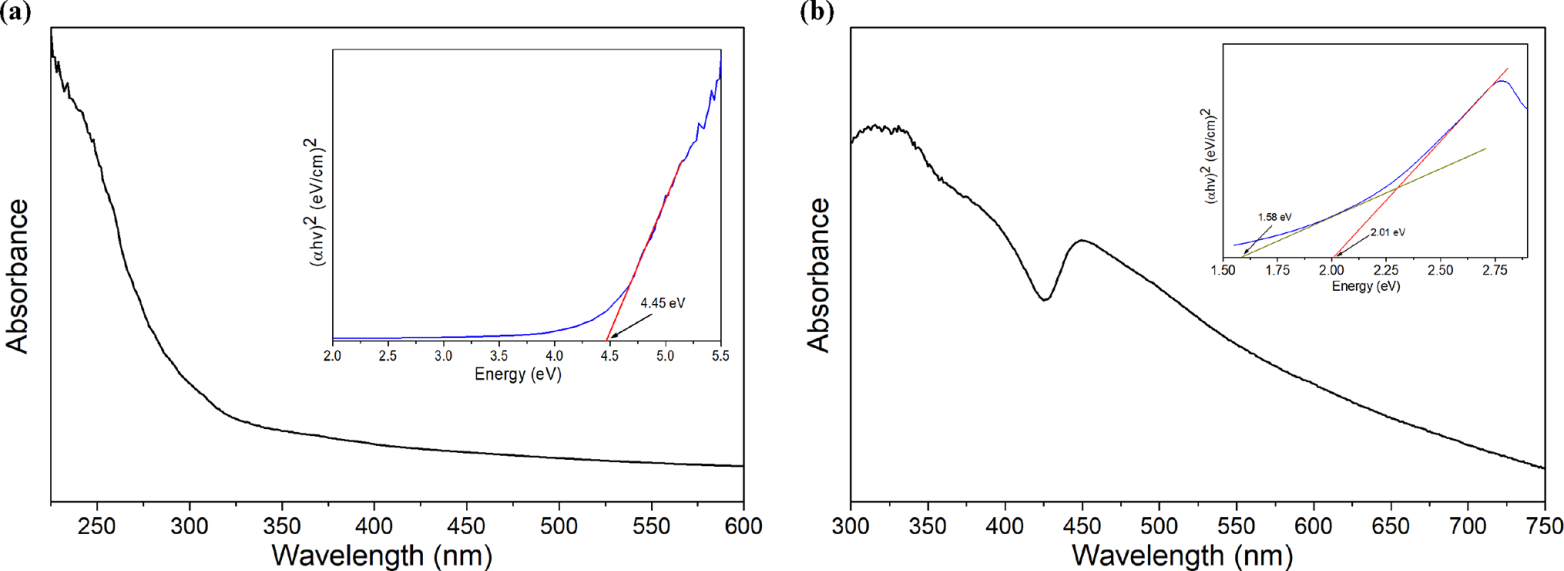
UV–visible absorption spectra and Tauc plots (insets) for Cs_3_Cu_2_Br_5_ (a) and Cs_2_AgBiBr_6_ (b) NCs. Sample preparation consists of suspending the NCs in isopropanol in a 10 × 10 mm fluorescence quartz cuvette. Subsequently, the cuvette was sealed and deaerated by purging with an argon gas stream for 15 min.

The UV–visible absorption spectra of the lead-free perovskite NCs were obtained using a Cary 5000 UV–vis–NIR spectrophotometer (Agilent), and subsequently, the band-gap energies were estimated through a Tauc plot. With regard to the Cs_3_Cu_2_Br_5_ NCs (Figure 2a), a well-known band edge absorption at about 260 nm with a long absorption tail at longer wavelengths is observed. Previously, density functional theory (DFT) calculations and density of states (DOS) analysis revealed that Cs_3_Cu_2_Br_5_ NCs present a direct band gap^21^ in which the valence and conduction bands are contributed by the Cu 3d orbitals and Cu 4s orbitals hybridized with the Br 5p orbitals, respectively. Therefore, the energy gap was determined by directly extrapolating the linear portion of the graph between the (α*hv*)^2^ function versus the photon energy (inset in Figure 2a). As a result, Cs_3_Cu_2_Br_5_ NCs revealed an experimental band gap of 4.45 eV in accordance with previous works.^17,21,22^

The absorption spectrum of the Cs_2_AgBiBr_6_ NCs exhibit an exciton peak at 450 nm (Figure 2b) and a long absorption tail to 750 nm, confirming the presence of sub-band-gap states.^19^ This perovskite shows a characteristic indirect band gap with a shallow absorption region followed by a sharp increase (inset in Figure 2b). Therefore, considering an indirect allowed transition, the Tauc plot reveals transitions at 1.58 and 2.01 eV, corresponding to the absorption and emission of a phonon.^23^ In consequence, the calculated indirect band gap is approximately 1.79 eV, which is in accordance with previous findings in the literature.^19,23^

Steady-state photoluminescence (PL) measurements were obtained using an Edinburgh Instruments FLS1100 spectrofluorometer and a 450 W Xenon lamp light equipped with a double monochromator coupled to a cooled photomultiplier (PMT-980). The optical properties were preliminarily studied under different atmospheres, such as of argon and oxygen. Additionally, NH_3_ and C_7_H_8_ were balanced in air to better understand the sensing mechanism.

### Detection of Gas Compounds

**Figure 3:**
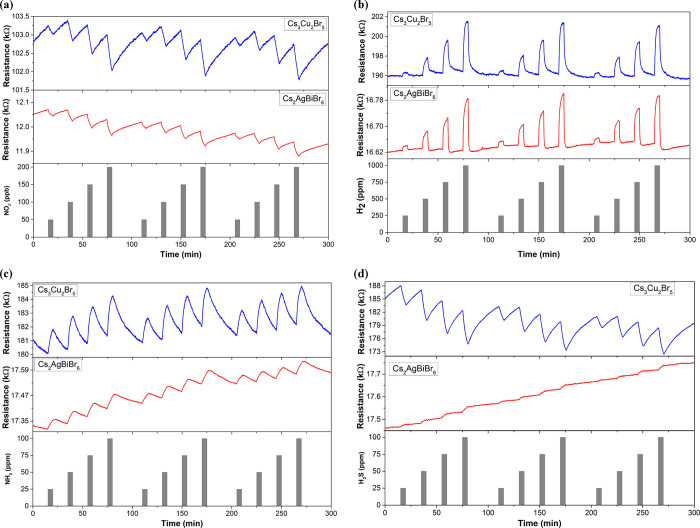
Example of electrical responses when detecting NO_2_ (a), H_2_ (b), NH_3_ (c), and H_2_S (d) at room temperature. All gases were tested in the ppm range except NO_2_, which was detected at ppb concentrations. Blue and red lines correspond to Cs_3_Cu_2_Br_5_ and Cs_2_AgBiBr_6_ supported on graphene.

The sensing performance of lead-free perovskites suspended on graphene toward different gas compounds was studied. Overall, increasing concentrations and repeated recovery steps were applied in similar conditions to those needed for real-time ambient monitoring. Figure 3a shows the dynamic resistance changes obtained for the two samples when detecting NO_2_ at parts per billion levels. Concentrations ranging from 50 to 200 ppb were applied, revealing higher responses (more than twofold) for the graphene hybrid with Cs_3_Cu_2_Br_5_ NCs (Figure S3a). As expected for a p-type sensitive layer, the resistance levels of both sensors decreased when exposed to an electron-withdrawing gas such as NO_2_, owing to the higher density of positive carriers. Both lead-free perovskite nanocrystals presented an ambipolar behavior, but the p-type behavior of the film was given by graphene, which is the most abundant material in the hybrid. Defining the sensor sensitivity as the slope of the calibration curves shown in Figure S4a, graphene decorated with Cs_3_Cu_2_Br_5_ perovskites showed about 2 times higher sensitivity than graphene loaded with Cs_2_AgBiBr_6_ (Table 1).

**Table 1:** Comparison of the Sensitivity, Limit of Detection (LOD), and Limit of Quantification (LOQ) for Both Lead-Free Nanocrystals Supported on Graphene when Detecting Several Gases

target gas	sample	sensitivity^a^	LOD	LOQ
NO_2_	Cs_3_Cu_2_Br_5_@graphene	7.57	8.5	28.2
	Cs_2_AgBiBr_6_@graphene	4.04	26.3	87.8
H_2_	Cs_3_Cu_2_Br_5_@graphene	3.08	24.4	81.5
	Cs_2_AgBiBr_6_@graphene	1.06	41.4	137.9
NH_3_	Cs_3_Cu_2_Br_5_@graphene	21.39	13.95	46.49
	Cs_2_AgBiBr_6_@graphene	1.24	43.52	145.08
H_2_S	Cs_3_Cu_2_Br_5_@graphene	75.44	13.6	45.3
	Cs_2_AgBiBr_6_@graphene	0.21	52.4	174.7

a Sensitivity expressed as % × ppb^–1^ for NO_2_ and % × ppm^–1^ for H_2_, NH_3_, and H_2_S gases (sensitivity is given by the slope of the calibration curves; the LOD and LOQ are expressed in ppb for NO_2_ and ppm for H_2_, NH_3_, and H_2_S).

More interestingly, for evaluating the potential application of the developed nanomaterials in real conditions, it is needed to estimate their detection and quantification limits (LOD and LOQ, respectively). Considering the experimental results obtained, these limits were estimated through the following equations:
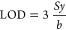
1
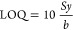
2where *S_y_* corresponds to the standard deviation of *y* residuals, while *b* is the sensitivity (slope) obtained from the calibration curve. As a result, the Cs_3_Cu_2_Br_5_ supported on graphene shows an LOD and LOQ of 8.5 and 28.2 ppb, respectively (Table 1). These NO_2_ limits are below the concentrations required for ambient monitoring applications. The U.S Environmental Protection Agency (EPA) defines the 1 h daily maximum NO_2_ concentration and the annual mean as 100 and 53 ppb, respectively.^24^ Similarly, the air quality standards defined by the European Commission establish 106 and 21 ppb as the maximum exposure for an average period of 1 h and a year, respectively.^25^ Despite the lower sensitivity of Cs_2_AgBiBr_6_, this nanomaterial shows an LOD and LOQ of 26.3 and 87.8 ppb, respectively (Table 1). It is worth mentioning that sensitivity, LOD, and LOQ values are representative of the experimental conditions applied. In this sense, these values would be further improved by optimizing the experimental setup and the operating conditions.

Not limited to this, acute exposure to higher concentrations of NO_2_ induce health problems such as eye irritation and irregulations in the respiratory system (e.g., cough, decreased pulmonary function, and dyspnea) and cardiovascular system such as tachycardia.^26^ Therefore, considering that the National Institute for Occupational Safety and Health (NIOSH) established 1 ppm of NO_2_^27^ as the recommended exposure limit (REL), several measurements were carried out at this concentration range. Increasing NO_2_ levels from 250 ppb to 1 ppm were tested (Figure S5a), revealing repeatable responses and better sensing performance for the Cs_3_Cu_2_Br_5_ NCs supported on graphene (Figure S5b). As expected, significant sensing responses were obtained when detecting NO_2_, owing to the large charge transfer between this gas with both graphene and lead-free perovskites.^28,29^ NO_2_ has a lone-pair electron, and when it is adsorbed, the bond distance is usually short, resulting in a relatively large charge transfer in comparison to gases of a different nature. It is worth noting that the bare graphene was also tested for detecting NO_2_ at this concentration range. However, Figure S5a demonstrates that their resistance changes are significantly lower in comparison to the graphene loaded with NCs. Additionally, Figure S5b shows that bare graphene presents a lower sensitivity (up to fivefold) than that of the nanocomposites developed. Therefore, considering the limited sensing performance of the bare graphene sensor, the use for detecting lower NO_2_ levels and other gases that induce lower responses is not shown.

Figure 3b shows the dynamic responses of both hybrids when detecting H_2_ in the 250–1000 ppm range. Specifically, four different hydrogen concentrations were applied, revealing repeatable responses. However, calibration curves depicted in Figure S4b show that Cs_3_Cu_2_Br_5_ presents higher responses and sensitivity (up to threefold) than those of Cs_2_AgBiBr_6_ NCs, resulting in lower detection and quantification limits (Table 1). Unlike NO_2_, hydrogen is not toxic, but concentrations above 4% can create explosive atmospheres.^30^ Nevertheless, both perovskites supported on graphene are capable of detecting and quantifying H_2_ concentrations far below the safety levels. It is worth noting that these measurements constitute the first time that lead-free perovskites are used for detecting hydrogen.

Another electron-donor gas compound such as NH_3_ was also tested. Different concentrations (ranging from 25 to 100 ppm) were applied (Figure 3c), revealing clear resistance changes when they are exposed to the analyte. However, it is worth highlighting that, while Cs_3_Cu_2_Br_5_ supported on graphene shows repeatable sensing responses and a slight resistance baseline drift, the Cs_2_AgBiBr_6_ NCs show a significant baseline drift and poor resistance changes. This fact reveals that Cs_2_AgBiBr_6_ experiences a lower charge transfer when interacting with NH_3_, but the binding energy should be relatively high, considering the drift experienced with the resistance baseline. Interestingly, Figure S4c depicts the calibration curves for both sensors. The Cs_2_AgBiBr_6_ presents an extremely low sensitivity (i.e., the slope of the curve), or in other words, increasing concentrations of NH_3_ induce negligible increases in the resistance changes. These results constitute an important barrier for employing the Cs_2_AgBiBr_6_ NCs for detecting this gas in ambient monitoring applications. Conversely, Cs_3_Cu_2_Br_5_ perovskites present clearer responses for the different NH_3_ levels and higher stability, resulting in higher sensitivity (17-fold than the Cs_2_AgBiBr_6_ NCs) and lower detection and quantification limits (Table 1). Considering that the NIOSH defines the recommended exposure limit (REL), the permissible exposure limit (PEL), and the immediately dangerous to life and health concentration (IDLH) as 25, 50, and 300 ppm of NH_3_, respectively,^31^ the Cs_3_Cu_2_Br_5_ NCs have been constituted as a potential candidate for their use in real sensing purposes. It is worth mentioning that, overall, sensing responses to NH_3_ and H_2_ are lower in comparison to NO_2_. According to theoretical calculations, these experimental findings can be expected owing to the small charge transfer between these gases and the active films,^28,32^ especially for room-temperature-operated gas sensors.

Finally, hydrogen sulfide (H_2_S) was measured for the first time using lead-free perovskites. In particular, four different concentrations (25, 50, 75, and 100 ppm) were applied during consecutive cycles (Figure 3d). This range of H_2_S levels comprises the REL, PEL, and IDLH levels (10, 50, and 100 ppm, respectively) defined by the NIOSH.^33^ The Cs_2_AgBiBr_6_ NCs supported on graphene have not been constituted as an alternative for detecting this gas compound since extremely low resistance changes and a continuous baseline drift (revealing an irreversible interaction) can be observed. In contrast, the Cs_3_Cu_2_Br_5_ perovskites demonstrated suitable H_2_S detection. Surprisingly, the sensitivity of Cs_3_Cu_2_Br_5_ is approximately 360 times higher than that of Cs_2_AgBiBr_6_ (Table 1), and its detection and quantification limits are below the threshold limit values for the H_2_S exposure. As expected, an electron-donor gas such as H_2_S should increase the resistance level of a mild p-type semiconductor such as Cs_2_AgBiBr_6_ supported on graphene. However, Cs_3_Cu_2_Br_5_ perovskites supported on graphene showed the opposite behavior (i.e., n-type semiconductor), decreasing their resistance levels when exposed to H_2_S. The reason for this change is still controversial, but the exothermic adsorption of H_2_S might be a plausible explanation. Considering the excellent response of Cs_3_Cu_2_Br_5_ toward H_2_S, this exceptional interaction would induce an intense exothermic process. Indeed, DFT calculations demonstrated that doped carbon-based nanomaterials can shift from a p-type to an n-type behavior because of the Seebeck effect when H_2_S is detected.^34^ This phenomenon might occur when an intense exothermic reaction through the H_2_S adsorption creates a temperature gradient in the sensitive layer that is converted into a thermoelectric effect.

It is worth mentioning that Cs_3_Cu_2_Br_5_ NCs supported on graphene show a significant experimental uncertainty, which is expressed as standard deviation (Figure S4d), probably because of the slight resistance baseline drift over time. The reason is that the interaction with H_2_S is partially irreversible due to the strongly reducing properties of this gas. In addition, the exothermic adsorption of H_2_S is translated into a thermoelectric effect that is probably lowering the repeatability of the sensing responses.

As a result, progressive desensitization is observed after each cycle of four concentrations. Nevertheless, unlike gases for ambient monitoring purposes (e.g., hourly fluctuations of NO_2_ levels), the H_2_S is more associated with leakage events. Additionally, considering that PEL exposure is defined as 50 ppm of H_2_S for 10 min, their detection and sensor reversibility were studied by applying those conditions (Figure S6). The clear and fast response registered is worth noting, and despite the sensor recovery process needing a few hours, the reversibility toward H_2_S is almost completed.

**Figure 4:**
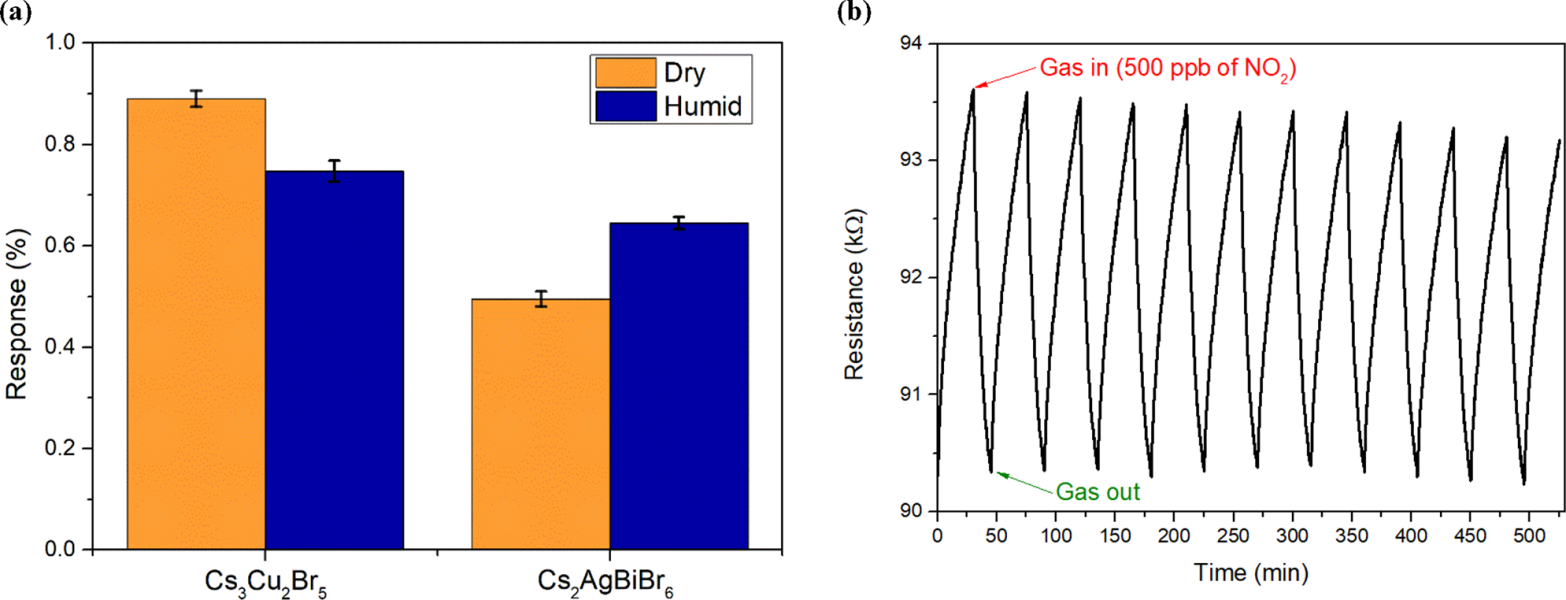
Comparison of the sensing responses at room temperature toward NO_2_ for Cs_3_Cu_2_Br_5_ and Cs_2_AgBiBr_6_ NCs supported on graphene under a dry and a humid (70% R.H.) environment (a). Repeatability test conducted via consecutive exposures of NO_2_ (500 ppb) for the Cs_3_Cu_2_Br_5_ NCs at room temperature (b).

Furthermore, the presence of ambient moisture when detecting gas compounds plays an essential role in the sensor instability or cross-interference. In this perspective, the NO_2_ gas was detected in a dry and humid (40% of relative humidity) environment by applying consecutive pulses of 200 ppb (Figure S7). Both types of lead-free perovskites can effectively detect NO_2_ in both atmospheric conditions and with outstanding repeatability. This fact demonstrates the high stability of the inorganic perovskites utilized toward the relative humidity. However, interestingly, as Figure 4a depicts, the presence of ambient moisture slightly decreases the response of Cs_3_Cu_2_Br_5_ NCs, while it increases that of Cs_2_AgBiBr_6_. The sensing mechanism involving lead-free perovskites in a humid environment is still controversial, but recently, it was reported that Cs_3_Cu_2_Br_5_ is not intensively affected at such relative humidity levels.^35^ In this perspective, water molecules can passivate the Cs_3_Cu_2_Br_5_ NCs since their interaction is competitive with NO_2_. When H_2_O molecules interact with this perovskite, a smaller charge transfer would occur in comparison to the target gas, resulting in a slightly lower response to NO_2_. Conversely, Cs_2_AgBiBr_6_ has been demonstrated to show high reactivity and excellent charge transfer when interacting with H_2_O molecules,^36^ which probably enhances the response in a humid environment. At 40% of relative humidity, chemisorption of water can be expected at the Cs_2_AgBiBr_6_, which does not compromise the charge transfer.^37^ However, at higher humidity levels, physisorption can be also expected over the chemisorbed water molecules, creating a sort of humidity layer over the sensitive film and inducing a proton hopping.^37^ To better apprehend the high potential of lead-free perovskite nanocrystals, additional sensing parameters were studied using the developed nanocomposites. The sensor repeatability (Figure 4b) was assessed by applying consecutive NO_2_ exposures (500 ppb) for 15 min and between them, recovery steps of 30 min using pure synthetic air. The Cu-based perovskite NCs supported on graphene have demonstrated outstanding air stability over time^38^ as well as noteworthy repeatability (relative error of 3.1%).

**Figure 5:**
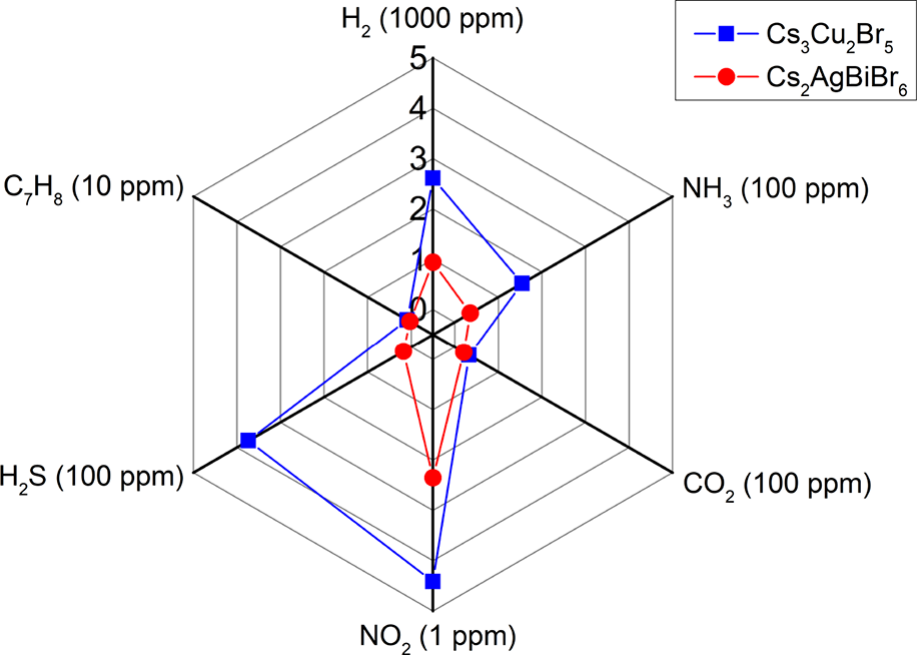
Radar graph summarizing the sensing responses for different target gases and comparing the performance of both lead-free perovskites employed.

Nevertheless, cross-sensitivity can be a significant drawback that can prevent the implementation of the developed nanocomposites in commercial devices. In this regard, additional gases such as toluene (C_7_H_8_) and carbon dioxide (CO_2_) were measured. Figure 5 summarizes the sensing responses toward different analytes. On the one hand, it can be observed that Cs_3_Cu_2_Br_5_ shows a higher signal than Cs_2_AgBiBr_6_ for all the gas compounds tested. On the other hand, considering the resistance changes with the concentration applied for each target gas, the NO_2_ detection stands out due to the higher sensing response obtained despite the lower concentration tested. In consequence, the Cs_3_Cu_2_Br_5_ NCs supported on graphene are a promising option for monitoring the NO_2_ levels in the atmosphere. Nonetheless, it is also worth highlighting the sensitive and reversible detection of H_2_S, being an alternative for detecting leakage events of this harmful gas compound.

### Gas Sensing Mechanisms

To date, the few works that reported the use of lead-free perovskites as gas sensors mostly employed them as bulky thin films at room temperature.^29,39^ However, the present work summarizes outstanding sensing performance probably because of the use of lead-free perovskites as nanocrystals. For understanding this concept, it is important to define an exciton, which is an excited pair (electron–hole) that experiences a mutual attraction to form a neutral quasiparticle. Thus, the exciton binding energy would possess a key role in sensing performance. When lead-free perovskites are employed to grow thin films and are operated on at room temperature, the exciton binding energy is smaller than the thermal energy.^40^ As a result, free carriers are generated but without transporting a net electric charge.^41^ Conversely, the present work reported the use of nanocrystals, which are confined structures that increase the exciton binding energy above the thermal energy under room-temperature operations. As a result of this confinement effect, the perovskite nanocrystals tend to promote the radiative recombination of carriers and suppress the exciton dissociation.^42^ In addition, perovskite nanocrystals can easily form self-trapped excitons that further promote the interaction with gas compounds.^43^ For that reason, supporting lead-free perovskite nanocrystals on graphene seems to be a suitable alternative for developing semiconducting-sensitive layers. In the first step, nanocrystals would intensively interact with the target gases. Additionally, if the perovskites are supported on graphene, it is possible to take advantage of the high and efficient carrier mobility of the graphene.

It is also worth noting the defect tolerance of halide perovskites, which is correlated to the lack of bonding–antibonding interactions between the valence band (VB) and the conduction band (CB).^44^ At the nanoscale, this property has special importance owing to the relatively high surface-to-volume ratio of the defects.^45^ Perovskites have been mostly used in optical applications in which defects are an adverse parameter that should be removed to the maximum extent. In contrast, the defects present in perovskites can be beneficial in semiconducting applications to some point because defects can serve as electronic traps, inducing a higher interaction with gas compounds. Additionally, lead-free perovskites possess worse optical properties than lead-based perovskites owing to their larger density of defects.^40^ However, once again, this high concentration of defects in lead-free perovskites is probably beneficial from the gas sensing point of view as observed in this work in comparison to lead-based perovskites.^9^ In this perspective, the perovskite defects, dangling bonds, and even the presence of organic ligands can form trap states^46^ that tend to increase the interaction with the target gases. This probably explains the excellent properties of perovskite NCs when employed in gas-sensing devices. It is also worth mentioning that, in perovskite nanocrystals, the CB consists of spin–orbit coupling, while the VB has an antibonding nature. These contrasting differences in the orbital characteristics induce the formation of shallow trap states,^47^ which probably further increases the sensitivity to the target gases.

The relative crystal momentum between the lowest level of the VB and the maximum level of the CB determines whether a band gap is direct or indirect. In other words, in a direct band gap, the absorption and recombination only involve photons, whereas in an indirect band gap, this process also involves assistant phonon transitions, which makes this process proceed at a much slower rate. In this perspective, considering semiconducting gas sensors, a direct band gap would result in a shorter response time than an indirect band gap.^48^ Therefore, for the same exposure time to target gases, higher resistance changes can be expected for a direct band gap, especially if the exposure time is short enough for not achieving the response saturation. This is crucial for ambient monitoring purposes because the pollutant levels usually are fluctuating over time. Thus, a sensor saturation operated at room temperature toward a specific gas usually requires a high concentration for a relatively long-term period. This fact is probably one of the reasons explaining the better sensing performance of Cs_3_Cu_2_Br_5_ than Cs_2_AgBiBr_6_ (perovskite NCs with direct and indirect band gaps, respectively) toward a wide variety of pollutants. This agrees with the experimental findings shown in the present work (Figure 3), especially considering the short exposure times applied and the dynamic detection of gases performed.

**Figure 6:**
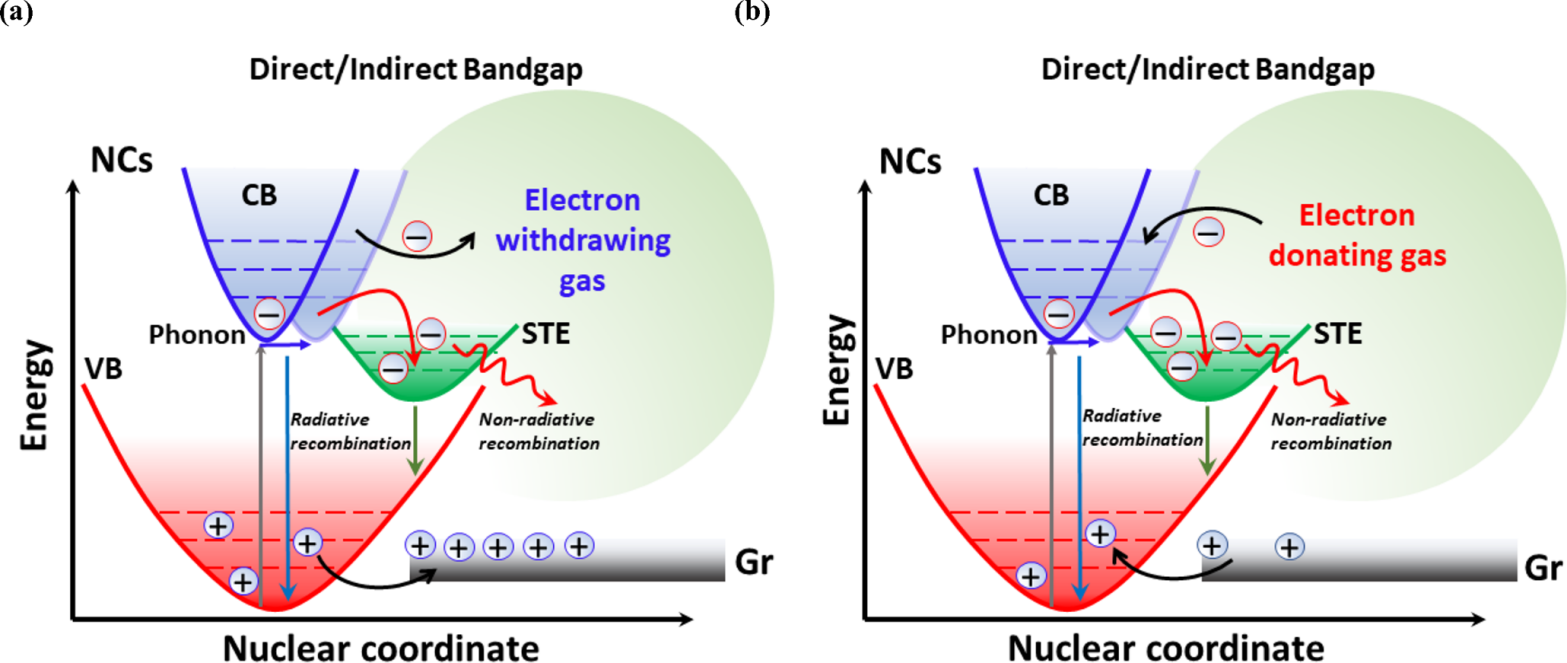
Proposed sensing mechanism for lead-free perovskites. Band-gap narrowing and radiative recombination of carriers through energy landscapes. Comprehensive detection mechanism for oxidizing (a) and reducing (b) gases when employing lead-free perovskite nanocrystals supported on graphene. STE: self-trapped excitons. Gr: graphene.

Another reason related to the band-gap narrowing of perovskite nanocrystals is the presence of self-trapped excitons when gas compounds interact with the lead-free perovskites inducing excited-state transitions, consisting of recombinations of free excitons whose emission energy is much smaller than the band gap. In other words, when the electrons are excited, they leave the ground state, inducing the formation of holes in that ground state. Afterward, electrons and holes radiatively recombine in pairs.^49^ It is worth mentioning that perovskites present multiple energy landscapes across the band gap, resulting in a concentration of carriers on smaller band gaps (Figure 6a). The defect tolerance of the synthesized perovskite NCs owing to the VB and CB formed by antibonding orbits^50^ leads to a high density of defects that act as self-electronic traps for significantly increasing the gas adsorption. This enhancement was previously reported in photocatalysis applications,^51,52^ and it can be also applied for gas sensing purposes. According to the type of semiconductor (direct or indirect), it has also been demonstrated that the strength of exciton–phonon coupling in direct band-gap NCs is moderate, favoring the radiative self-trapped excitons. In contrast, indirect band gaps can show a strong carrier–phonon coupling effect, resulting in more effective non-radiative self-trapped states. This again suggests the importance of the type of semiconductor and the strength of exciton–photon coupling in the sensing mechanism. Therefore, the direct and indirect band gap also influences the properties of the free exciton, the strength of exciton–phonon coupling, and the radiative or non-radiative nature of self-trapped excitons, determining the degree of interaction with the gas molecules.^53^ In this regard, to give some insight into the proposed process, the changes in the photoluminescence (PL) of the supported NCs under different gases were studied. Although these experiments still require further study, it is clearly observed that the magnitude of the PL changes under different gases. This change is much larger in the case of Cs_3_Cu_2_Br_5_ than Cs_2_AgBiBr_6_ (Figure S8). These results support the importance of the strength of the exciton–phonon coupling in the sensing mechanism.

Nevertheless, the comprehensive sensing mechanisms in lead-free perovskite nanocrystals supported on graphene involve both nanomaterials. In this regard, despite the fact that a direct interaction of graphene with the gas compounds cannot be ruled out, considering the low sensitivity of bare graphene, the charge transfer is probably negligible in comparison to the nanocrystals. In other words, a limited interaction and small charge transfer can be expected between the target gases and the oxygen-containing functional groups grafted at the graphene surface. However, the presence of lead-free perovskite nanocrystals has a crucial role in enhancing the sensing performance, increasing the sensitivity, and lowering the detection and quantification limits. In the nanohybrids developed, graphene would act as a transducing element owing to its excellent carrier transport. For that reason, while perovskite nanocrystals are ambipolar charge carriers, the sensitive film usually acts as a p-type semiconductor because graphene is the dominant carrier transport nanomaterial.

In this perspective, when detecting oxidizing gas compounds (i.e., electron-withdrawing), the resistivity of the film decreases owing to the higher density of positive carriers (holes) in graphene, which is the transducing element. For instance, when NO_2_ interacts with the NCs, the electron–hole pairs are separated and can be moved toward energy landscapes across the band gap. The interaction of an electron-withdrawing gas with the perovskites induces an accumulation of positive charge carriers at the nanocrystals (Figure 6a). Then, an interface between graphene and the perovskite NCs is generated for compensating the carriers at the lead-free perovskites. As a result, the graphene layer, which is the transducing element and the main charge carrier transporter, possesses an excess of holes (i.e., majority of charge carriers in a p-type material), further decreasing the resistance level of the film. Conversely, when an electron-donor gas (e.g., NH_3_) interacts with the lead-free perovskites, negative carriers are accumulated at the nanocrystal (Figure 6b). Therefore, graphene would transfer positive carriers over the energy barrier from its VB to the perovskites. This process results in an excess of electrons in the graphene layer. In consequence, the presence of perovskite NCs further increases the resistivity of the layer since negative carriers are not the majority of charge carriers in p-type semiconductors. An analogous mechanism has been previously discussed in lead halide perovskites NCs.^9,10^ Therefore, considering the high reactivity of lead-free perovskites and the excellent charge transport of graphene, high sensing responses were obtained as expected. It is worth highlighting the band-gap narrowing in perovskite nanocrystals due to the presence of self-trapped states because this effect probably enables a more efficient transport of charge carriers between the graphene and the nanocrystals. In this perspective, the Cs_3_Cu_2_Br_5_ (direct bandgap) probably has lower energy barriers than Cs_2_AgBiBr_6_ (indirect bandgap) with respect of graphene energy levels. This can be translated into a more efficient extraction of charge carriers of Cs_3_Cu_2_Br_5_ NCs and, in consequence, higher sensing responses.

## Conclusions

Environmentally friendly and nontoxic nanomaterials such as lead-free halide perovskite nanocrystals were supported on graphene for detecting a wide variety of harmful gases at trace levels. In addition, the nanomaterials employed for developing the gas sensors are quite abundant and inexpensive, paving the way toward a straightforward and mass-scalable synthesis. Four different gases were effectively detected in a few minutes and at concentrations below the threshold limit values, revealing limits of detection and quantification low enough for ambient monitoring applications. In addition, remarkable stability and repeatability were achieved under room-temperature conditions, which paves the way for achieving low-power consumption devices.

The present work reports for the first time the development of a hybrid comprising graphene and lead-free perovskite nanocrystals. Additionally, some gases such as H_2_ and H_2_S were detected using lead-free perovskites for the first time, and the ultrasensitive detection of NO_2_ was achieved at parts per billion levels. To increase our knowledge, the intrinsic properties of the nanocrystals were studied for unraveling the sensing mechanisms and correlating them with significant differences observed in the sensing performance. Overall, the Cs_3_Cu_2_Br_5_ NCs present higher sensitivity towards gase than the Cs_2_AgBiBr_6_ NCs, showing lower LOD and LOQ values for all the gases tested. Two main reasons can probably explain these experimental findings. On the one hand, the UV–vis absorption revealed that Cs_3_Cu_2_Br_5_ presents a direct band gap, which is favorable from the gas sensing point of view. Conversely, the Cs_2_AgBiBr_6_ NCs showed an indirect band gap. On the other hand, the strength of exciton–phonon coupling of self-trapped excitons in both types of perovskites NCs plays an important role, being stronger in the case of Cs_2_AgBiBr_6_ NCs with an indirect band gap, limiting the efficient generation of separate charges and reducing the interaction with gas molecules.

It is worth highlighting that all-inorganic-based perovskites such as the Cs_3_Cu_2_Br_5_ NCs usually present outstanding stability in comparison to organic–inorganic hybrid perovskites. The reason is that organic cations such as formamidinium or methylammonium are susceptible to degradation or decomposition under standard environmental conditions or when exposed to specific gas compounds. Additionally, considering the same experimental conditions and 5 min of gas exposure to NO_2_, the Cs_2_AgBiBr_6_ NCs supported on graphene show significantly better sensing performance than more harmful alternatives such as lead halide perovskites. For instance, the Cs_2_AgBiBr_6_ NCs present higher sensitivity (almost twofold) than lead-based NCs such as CH_3_NH_3_PbBr_3_^10^ when both are supported on graphene. This is probably because lead-free perovskites possess a defect tolerance and a high density of surface defects that are acting as electronic traps. These defects are probably a drawback in optical applications, but from the gas sensing point of view, since defects act as electronic traps for interacting with gas compounds, their presence is an opportunity for developing highly sensitive nanomaterials. Not limited to this, a wide variety of lead-free perovskite compositions can be developed, paving the way toward the exploitation of multiple configurations for selectively detecting the target gases.

## Supporting Information Available

Experimental section, histograms, HR-TEM, FESEM, calibration curves, NO_2_ detection (250–1000 ppb range), H_2_S detection, comparison of results under a dry and a humid atmosphere, and photoluminescence measurements (PDF)
