# Supplementary material for: Unraveling the Gas-Sensing Mechanisms of Lead-Free Perovskites Supported on Graphene
Source: ACS Sens. 2022 Nov 21;7(12):3753–63. doi: 10.1021/acssensors.2c01581 (PMC9791682; doi:10.1021/acssensors.2c01581)
Supplement: Supplementary file 1 — se2c01581_si_001.pdf [file se2c01581_si_001.pdf]

UNRAVELING THE GAS SENSING MECHANISMS OF LEAD-FREE PEROVSKITES SUPPORTED ON GRAPHENE

Juan Casanova-Chafer, [a*] *Rocío Garcia-Aboal,* [b*] *Pedro Atienzar,* [b] *Eduard Llobet* [a]

[a] MINOS Research Group, Department of Electronics Engineering, Universitat Rovira i Virgili, 43007 Tarragona, Spain.

[b] Instituto de Tecnología Química (Universitat Politècnica de València – Consejo Superior de Investigaciones Científicas), Avd. de los Naranjos s/n, 46022 Valencia, Spain.

Table of contents

1. Histograms..2
2. HR-TEM characterization..2
3. FESEM characterization..3
4. Gas sensing calibration curves..4
5. Detection of NO_2 at 250-1000 ppb range..5
6. H_2S detection..5
7. Comparison of resistance changes in dry and humid atmosphere..6
8. Photoluminiscence measurements..6

1. Histograms

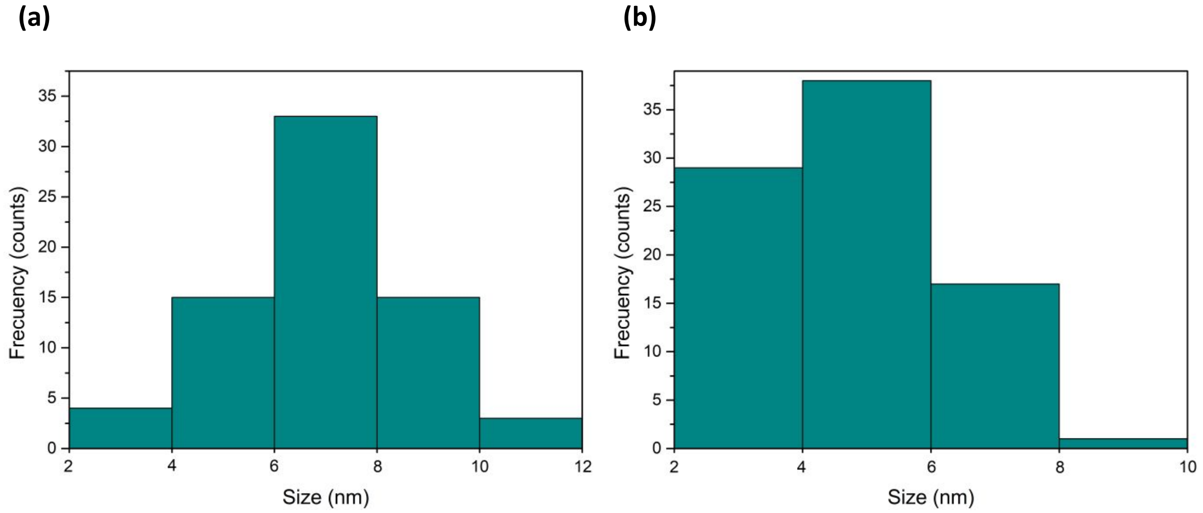

Figure S1. Histogram of nanocrystals size distribution for $Cs_3Cu_2Br_5$ (a) and $Cs_2AgBiBr_6$ (b) perovskites.

2. HR-TEM characterization

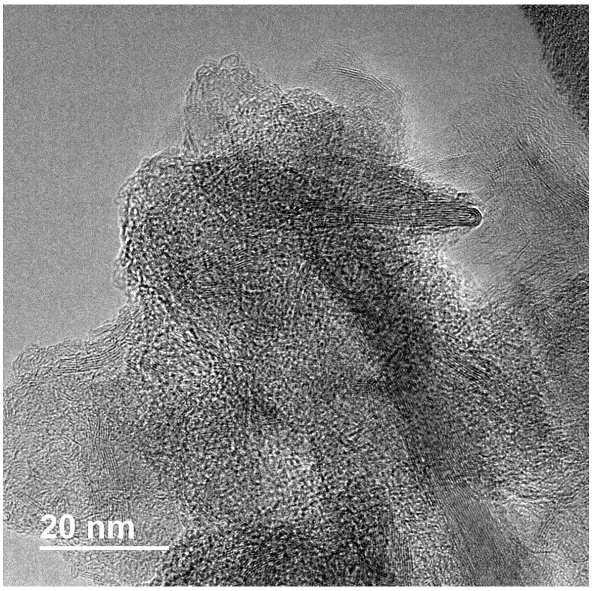

Figure S2. HR-TEM image for bare graphene nanoplatelets.

3. FESEM characterization

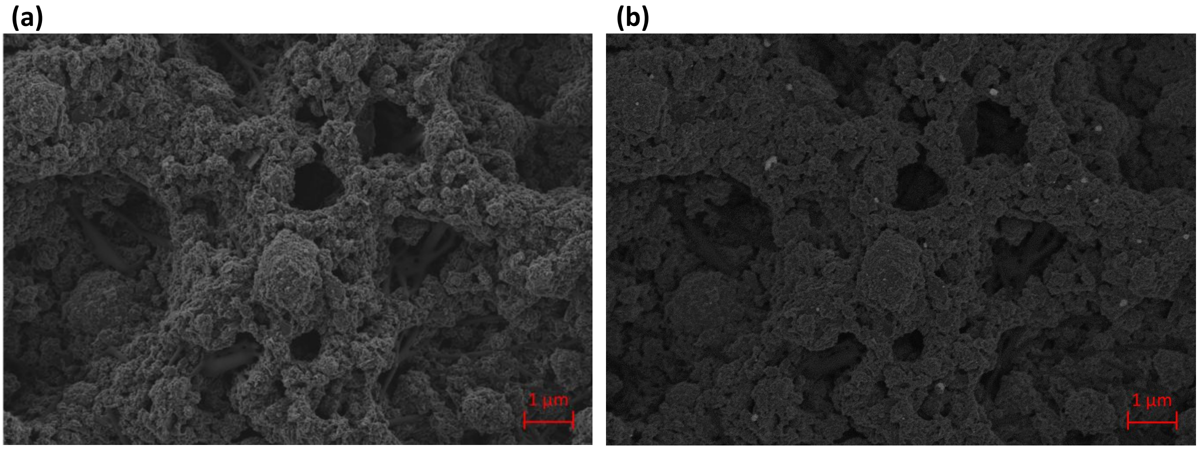

Figure S3. Example of FESEM image showing the sensor surface (**a**). FESEM image recorded over the same region of the sample but using the Back-scattered Electron Detector (BSE). Bright spots reveal the presence of superficial lead-free perovskites whereas black background corresponds to the graphene (**b**).

4. Gas sensing calibration curves

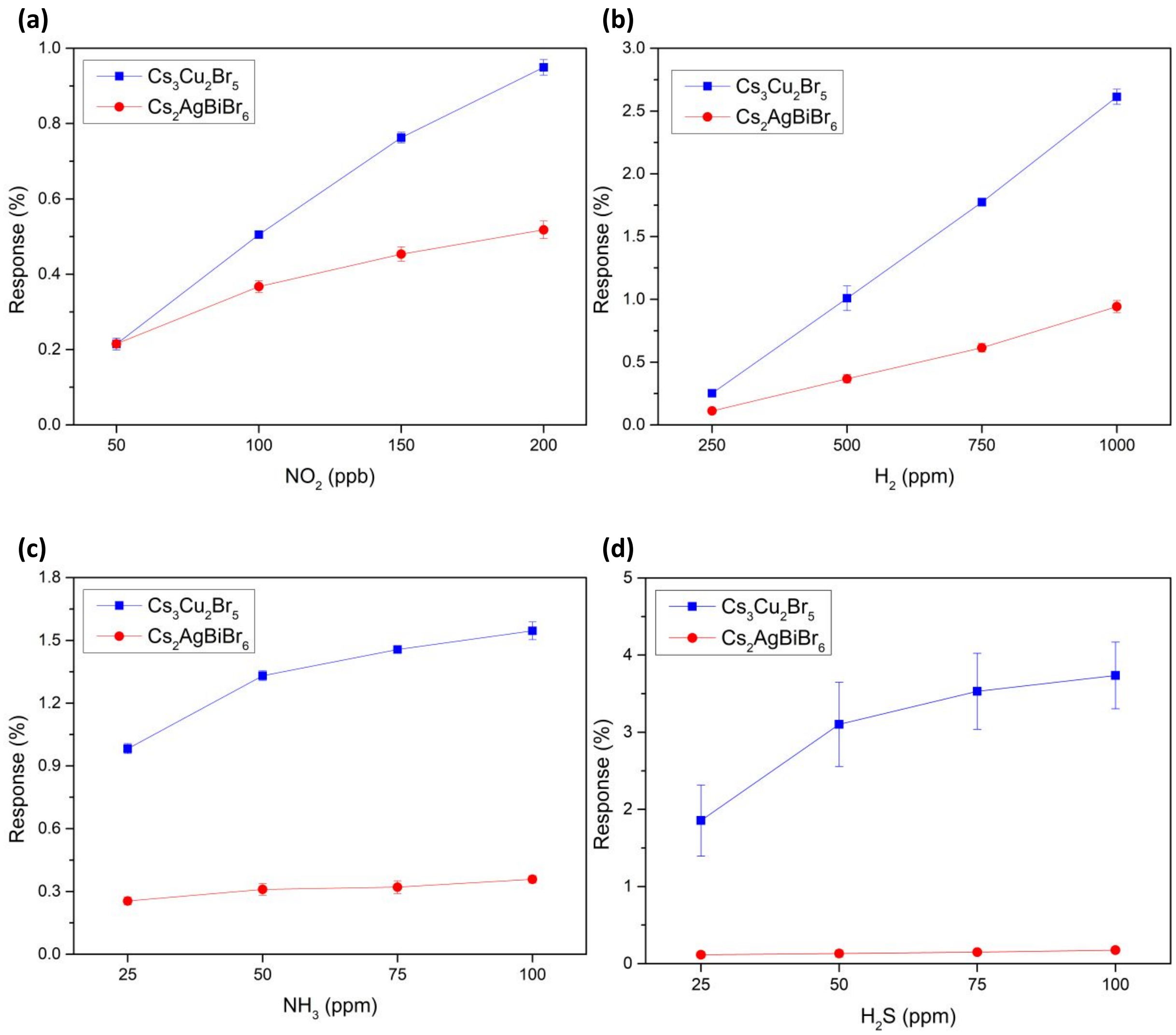

Figure S4. Comparison of calibration curves when detecting NO_2 (**a**), H_2 (**b**), NH_3 (**c**), and H_2S (**d**) at room temperature. Blue and red lines respectively correspond to $Cs_3Cu_2Br_5$ and $Cs_2AgBiBr_6$ supported on graphene.

5. Detection of NO_2 at 250-1000ppb range

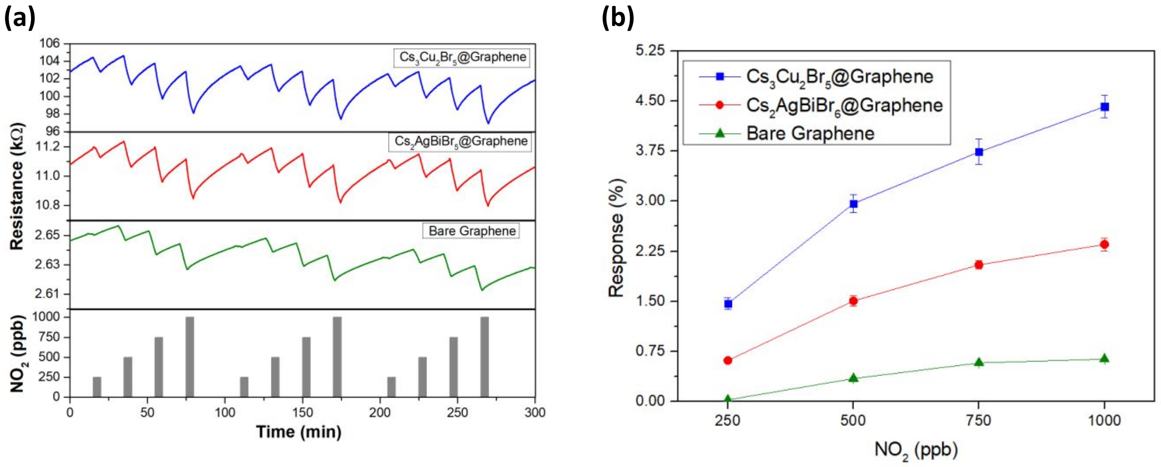

Figure S5. Example of resistance changes obtained when detecting NO_2 at the 250-1000 ppb range and at room temperature (**a**). Comparison of the calibration curves for pristine graphene and both types of lead-free perovskite nanocrystals supported on graphene (**b**).

6. H_2S detection

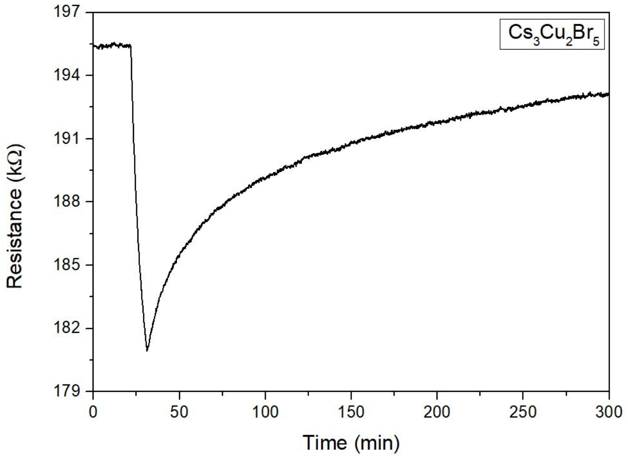

Figure S6. Example of PEL exposure to 50 ppm of H_2S for 10 minutes. The $Cs_3Cu_2Br_5$ nanocrystals supported on graphene effectively detect this harmful gas and the sensor was almost recovered after a few hours.

7. Comparison of resistance changes in dry and humid atmosphere

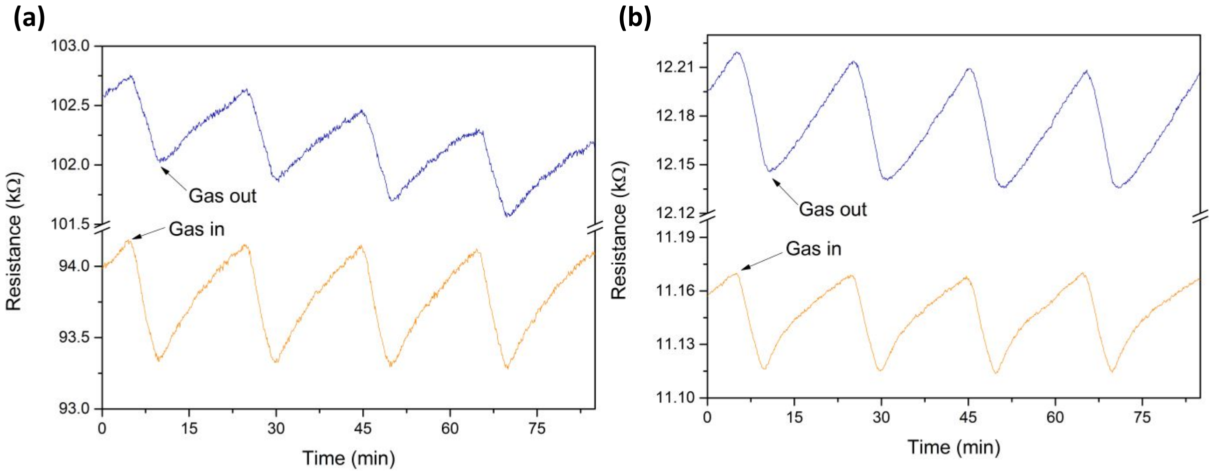

Figure S7. Comparison of the resistance changes obtained when detecting 200 ppb of NO_2 in a dry and a humid atmosphere for $Cs_3Cu_2Br_5$ (**a**) and $Cs_2AgBiBr_6$ (**b**) NCs supported on graphene.

8. Photoluminiscence measurements

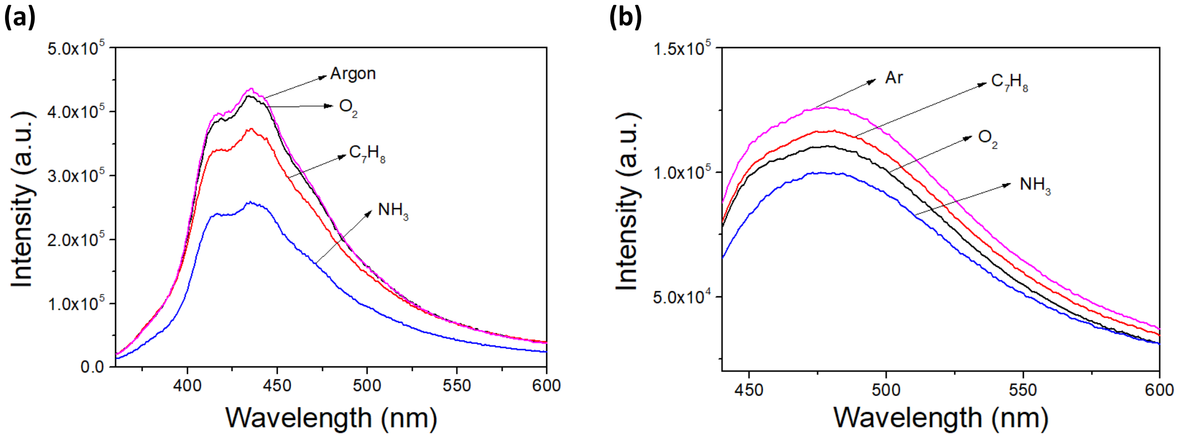

Figure S8. Room-temperature PL spectra of $Cs_3Cu_2Br_5$ (λ_{ex} = 340 nm) (a) and $Cs_2AgBiBr_6$ (λ_{ex} = 410 nm) (b) NCs supported on graphene when exposed under Ar, O_2, C_7H_8 and NH_3.
